# Longitudinal associations between body mass index and changes in disease activity and radiographic progression in rheumatoid arthritis patients treated with infliximab

**DOI:** 10.1136/rmdopen-2023-003396

**Published:** 2023-10-06

**Authors:** Theresa Burkard, Enriqueta Vallejo-Yagüe, Kim Lauper, Axel Finckh, Thomas Hügle, Andrea M Burden

**Affiliations:** 1 Department of Chemistry and Applied Biosciences, Eidgenossische Technische Hochschule Zürich, Zurich, Switzerland; 2 University of Bern, Institute of Primary Health Care (BIHAM), Bern, Switzerland; 3 Department of Rheumatology, University of Geneva, Geneva, Switzerland; 4 Department of Rheumatology, University Hospital Lausanne, Lausanne, Switzerland

**Keywords:** infliximab, rheumatoid arthritis, Disease Activity

## Abstract

**Objectives:**

Treatment response is worse in obese patients with rheumatoid arthritis (RA), including patients on weight-adjusted therapies like infliximab. We aimed to assess the association between body mass index (BMI) and changes in RA disease activity and radiographic progression over time.

**Methods:**

We included infliximab users with an RA diagnosis in the Swiss Clinical Quality Management in Rheumatic Diseases registry (1997–2020). Two cohorts were defined: (1) starting from their first BMI measurement or disease activity score (DAS28-esr), and (2) from their first BMI measurement or radiographic assessment (Rau score). We evaluated the coefficient and 95% CI of BMI with changes in mean DAS28-esr (cohort 1) and mean Rau scores (a structural joint damage score, cohort 2) using generalised estimation equations, overall and stratified by BMI categories.

**Results:**

Cohort 1 comprised 412 patients (74% women, mean age 53 years, mean BMI 25). We observed no change in mean DAS28-esr with increasing BMI overall (adjusted coefficient: 0.00, 95% CI −0.02 to 0.02), or in BMI categories. Cohort 2 comprised 187 patients highly alike to those in cohort 1. We observed a significant decrease of 1.05 in mean Rau scores for every increase in BMI unit (adjusted coefficient: −1.05, 95% CI −1.92 to −0.19). Results remained statistically non-significant across BMI categories.

**Conclusions:**

Our longitudinal investigation suggests that BMI increase may not lead to changes in DAS28-esr in patients receiving infliximab, despite the weight-adapted dose. Yet, there may be a decrease in erosions with increasing weight non-limited to obese patients.

**WHAT IS ALREADY KNOWN ON THIS TOPIC**

- Overweight and obese patients with rheumatoid arthritis have increased disease activity but decreased radiographic joint damage.

**WHAT THIS STUDY ADDS**

- We have performed longitudinal analyses using continuous variables of body mass index (BMI), disease activity and radiographic joint damage to overcome methodological issues of previous cross-sectional analyses and of those analyses using binary variables.
- Our results suggest that among users of weight-dosed infliximab disease activity is not affected by BMI, and that radiographic joint damage is positively affected by increasing BMI.

**HOW THIS STUDY MIGHT AFFECT RESEARCH, PRACTICE OR POLICY**

- Our results suggest a paradigm shift during dose adapted infliximab treatment. While BMI increase may not lead to changes in disease activity, a potential protective effect of increasing BMI is not limited to the overweight and obese population.

## Introduction

Rheumatoid arthritis (RA) is a heterogenous auto-immune disease characterised by synovial inflammation, fluctuating disease activity over time, and the possibility for disability in untreated or difficult-to-treat disease.^1 2^ Furthermore, it is known that the prevalence of obesity and overweight is higher among patient with RA than in the general population.^3 4^ This is especially relevant for treating physicians because overweight and obese RA patients using biologic (b) or targeted synthetic (ts) disease-modifying antirheumatic drugs (DMARDs), were reported to be less likely to achieve minimal disease activity, depending on the drug mechanism.^5^ For example, both obesity and overweight were associated with lower than expected response to tumour necrosis factor inhibitors (TNFi),^5^ and in particular among women.^6^ Moreover, the response to the only TNFi which is dosed according to body weight, infliximab, was also hindered by high body mass index (BMI).^7–9^ Additionally, although disease activity is expected to drive radiographic joint damage, it is puzzling that obese RA patients have increased disease activity measurements and disability scores but decreased radiographic joint damage.^10^

However, most studies to date have not assessed longitudinal associations between weight and disease activity or radiographic progression but took a cross-sectional approach missing temporality and potentially causality.^11^ Particularly, longitudinal analyses and studies with continuous measures (instead of categorical variables) are missing.^7 12^

We hypothesise that assessments over time, allowing to analyse changes in both BMI and RA outcomes, may hold insights into their associations. Thus, this study aimed to investigate the association between changes in BMI and changes in RA disease activity and radiographic progression during infliximab treatment courses. The inclusion of infliximab only yields a homogeneous study population and an analysis independent of bDMARD agent and dose (which may confound or mediate the tested associations).

## Methods

### Patient and public involvement statement

Neither the patient nor the public was involved in this study.

### Study design and data source

We conducted a cohort study using data from the Swiss Clinical Quality Management in Rheumatic Diseases (SCQM) registry. The nationwide SCQM registry was established in 1997 and is used to prospectively follow RA patients during routine clinical practice in Switzerland.^13^ RA diagnoses are made by board-certified rheumatologists. Patients participating in SCQM come from a wide range of settings (ie, private practices, academic centres). Data capture include demographics, life-style factors, regular physical examination (ie, tender/swollen joint count), laboratory tests (ie, erythrocyte sedimentation rate (esr), C reactive protein (crp)), and patient-reported surveys (eg, Health Assessment Questionnaire). Patient information is updated at least once per year, or every time a patient has a change in antirheumatic therapy (captured by physicians who enter start and stop dates implying the last administration). Additional information on data standardisation and harmonisation can be found in online supplemental material 1.

10.1136/rmdopen-2023-003396.supp1 Supplementary data

### Study population

The study included RA patients from the SCQM registry between inception in 1997 and December 2020 during their continuous infliximab use. Based on our observations in SCQM, infliximab was administered with a dose interval mode of approximately 8 weeks. Thus, continuous infliximab use was defined from the start date until 61 days (further referred to as grace period) after the recorded stop date. Re-start of infliximab within this grace period was considered as treatment continuation. Moreover, patients with a follow-up of ≤90 days were excluded due to lack of follow-up.

**Figure 1:**
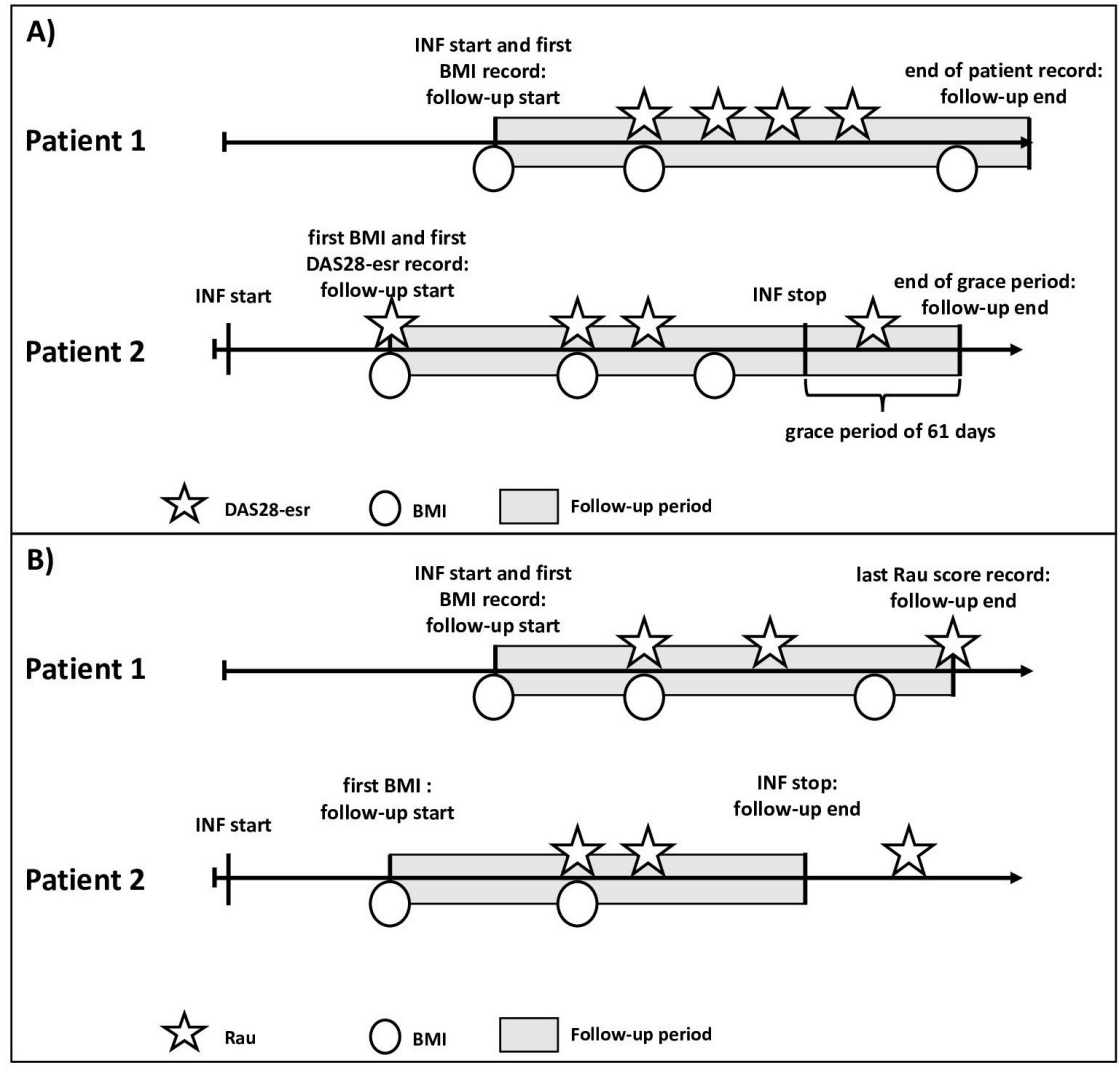
(A) Patient examples of cohort 1. Patients enter the cohort during infliximab use on the first date they have at least a BMI or DAS28-esr record. Patients are followed up until discontinuation of infliximab, end of patient record or end of study period, whichever occurred first. (B) Patient examples of cohort 2. Patients enter the cohort during infliximab use on the first date they have at least a BMI or Rau score record. Patients are followed up until discontinuation of infliximab or their last Rau score record. BMI, body mass index; DAS28-esr, rheumatoid arthritis disease activity measurement using 28 joints and erythrocyte sedimentation rate; INF, infliximab.

From the above-described population, we extracted two distinct cohorts, which were studied separately because of different study outcomes. Patient examples of the two cohorts are displayed in figure 1. First, to assess the association between BMI and disease activity (DAS28-esr) changes, cohort 1 included all patients who had at least two BMI and two DAS28-esr records during their continuous infliximab use. Cohort entry was defined as the day with the first BMI or DAS28-esr measurement.

Second, to assess the association between BMI and radiographic progression changes, cohort 2 included all patients who had at least two BMI and two Rau score records during their continuous infliximab use. Cohort entry was defined as the day with the first BMI or Rau score measurement.

### Exposure

The exposure of interest was BMI, captured as a continuous variable in kg/m^2^ at every available visit during infliximab use. Visits missing information on BMI were completed with the nearest BMI record within a 90-day range from the respective visit. Remaining missing values on BMI (7.0% in cohort 1, and 10.2% in cohort 2, online supplemental material 2) were imputed using multiple imputation by chain equation in a two-level approach (clustering by patient) which considers repeated measures for each patient.

### Outcomes

In cohort 1, our outcome of interest was DAS28-esr, captured as a continuous variable at every available visit during infliximab use. DAS28-esr was chosen because it is the most complete measurement of RA disease activity in SCQM. Missing DAS28-esr records were replaced by DAS28-crp records, if available. Subsequently, missing DAS28-esr values were complete within a 30-day range with existing values from the nearest visit. Remaining missing values (8.3%, online supplemental material 2) were imputed in the same run as the exposure.

**Figure 2:**
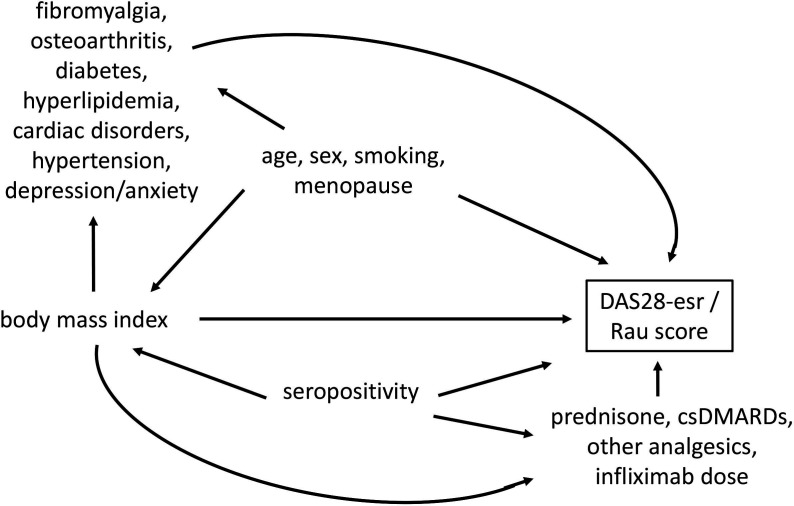
Directed acyclic graph (DAG) of the association between body mass index and DAS28-esr, and Rau score. Covariates potentially equally affect DAS28-esr and Rau scores, however, DAS28-esr as a proxy for RA disease activity is supposed to have a strong influence on the radiographic progression (ie, Rau score) and lies therefore on the causal pathway between BMI and radiographic progression. Covariates that lie on the causal pathway are called mediators. Comorbidities as well as RA medication related information are further considered mediators. BMI, body mass index; csDMARD, conventional synthetic disease-modifying antirheumatic drug; DAS28-esr, rheumatoid arthritis disease activity measurement using 28 joints and erythrocyte sedimentation rate; INF, infliximab; RA, rheumatoid arthritis.

In cohort 2, our outcome of interest was radiographic damage, scored using the Rau score, captured as a continuous variable at every available visit during infliximab use. The Rau score presents a semi quantitative method to score surface destruction in 38 joints of the hand, wrist and foot and go from 0 to 132.^14^ Missingness in Rau score were addressed with the nearest observation within a 30-day range. Remaining missing values (29.4%, online supplemental material 2) were imputed in the same run as the exposure. In this cohort, to prevent reverse causality, that is, falsely associating changes in Rau score to changes in BMI, we replaced each Rau score with the value of its subsequent visit. This helped to ascertain the direction of the associations according to the directed acyclic graph (DAG) (figure 2).

### Covariates

Covariates of interest included the following continuous variables: age, infliximab dose (as a proxy for disease severity), follow-up time, DAS28-esr measurement (cohort 2 only), prednisone dose (sensitivity analysis); and the following binary variables: sex, rheumatoid factor (RF) (we disregarded anti-citrullinated protein antibodies due to collinearity with RF and higher missingness than RF), smoking, alcohol consumption, csDMARD use, prednisone use, other pain/anti-inflammatory drug use, whether the patient was postmenopausal or had the following comorbidities: fibromyalgia, osteoarthritis, hypertension, diabetes, hyperlipidaemia, cardiac disorders, depression/anxiety (figure 2). Not all covariates made it into the final model which needed to converge (alcohol consumption was dropped because of high missingness, use of csDMARDs because of high prevalence, and diabetes and hyperlipidaemia because of collinearity with hypertension). Further information on lookback windows and whether the variables were used as time-varying can be found in online supplemental material 3.

In both cohorts, we replaced the value of the variables related to disease severity (infliximab dose and all other RA medication use) with the value of the subsequent visit because they were likely the result of the current DAS28-esr measurement rather than a potential cause (figure 2).

### Follow-up

In cohort 1, we followed all patients until discontinuation of infliximab (ie, treatment course plus grace period), end of patient record, or end of study period (December 2020), whichever occurred first. In cohort 2, because radiographic assessment in RA patients occurs infrequently,^15^ we followed all patients until discontinuation of infliximab or the last available radiographic assessment to not prolong the follow-up without additional outcome information, whichever occurred first.

### Data analysis

Except for the outcome definition across the two cohorts, identical statistical analyses were performed. We described the patient populations of each cohort for all variables of interest. Post hoc, we plotted the trajectories of BMI, DAS28-esr and of Rau scores of the patient population to visually depict changes in those variables over the follow-up time. We evaluated the coefficient and 95% CI of changes in BMI with the outcome mean DAS28-esr changes (cohort 1) and mean Rau score changes (cohort 2) using generalised estimation equations (GEE). This was done independently for each cohort overall, as well as stratified by BMI categories at cohort entry (or the first available measurement thereafter). These BMI categories were defined as underweight (<18.5 kg/m^2^), normal weight (BMI 18.5–24.9 kg/m^2^), overweight (BMI 25–29.9 kg/m^2^) and obese (BMI ≥30 kg/m^2^). In a sensitivity analysis, we further stratified the overall population by prednisone use at index date to assess a potential effect modifier effect of prednisone use.

The interpretation of the coefficient in GEE is similar to that in linear regression with the difference that GEE estimates the population average effect and thus compares changes in the mean outcome.^16^ In detail, the coefficient of the exposure (ie, BMI) implies the mean increase or decrease of the outcome per 1 unit increase in BMI. A coefficient of 0.00 means no change. A 95% CI that includes 0.00 implies a non-significant result.^17^ We assessed all associations crude and with increasing number of adjusting covariates to explore their influence on the model (DAG in figure 2). Our main model adjusted for infliximab dose, age, sex and follow-up time which were considered the most important variables without an overfitting risk. We used the Quasi Information Criterion to evaluate model fit and included interaction terms and squared/cubic terms where needed.^18^ Adjustment for continuous prednisone dose instead of binary prednisone use was added as a sensitivity analysis to account for the characteristics of prednisone leading to weight gain and decrease in RA disease activity. All analyses were carried out in STATA V.16 except for the multiple imputation which was carried out in BLIMP software V.2.2.^19^

## Results

Among 1153 RA infliximab users available in SCQM until 2020, 412 patients were included in cohort 1 and 187 patients in cohort 2 (flow chart in online supplemental material 4). Patient characteristics at cohort entry, separated by cohort, are described in table 1. In cohort 1, when assessing the longitudinal association between BMI and DAS28-esr, included patients had a minimum of two and a maximum of 27 observations (mean 6.4) during a mean follow-up of 4.5 years. For 82% of patients, the index date coincided with the first infliximab use. Most patients were women (74%) with a mean age of 53.4 years, mean BMI of 25.0, and mean DAS28-esr of 4.0. In cohort 2, when assessing the longitudinal association between BMI and Rau scores, included patients had a minimum of two and a maximum of 20 observations (mean 6.0) during a mean follow-up of 4.4 years. Patient characteristics were highly similar to those in cohort 1. Patients had a mean Rau score of 36. For 79% of patients, the index date coincided with the first infliximab use. Online supplemental material 5 depicts trajectories of BMI, DAS28-esr and of Rau scores which suggest substantial variability of those variables among the follow-up of the study population.

**Table 1:** Patient characteristics at cohort entry in the overall populations of cohort 1 (outcome: DAS28-esr) and cohort 2 (outcome: Rau scores)

	Cohort 1 412 patients (2646 visits)	Cohort 2 187 patients (1121 visits)
Mean follow-up time (SD) (years)	4.5 (4.1)	4.4 (3.6)
Median prevalent infliximab use (p25, p75) (days)	0 (0, 0)	0 (0, 0)
Mean age (SD) (years)	53.4 (12.6)	54.0 (11.6)
Women (%)	305 (74.0%)	145 (77.5%)
Men (%)	107 (26.0%)	42 (22.5%)
RF positive (%)	303 (73.5%)	150 (80.2%)
RF negative (%)	97 (23.5%)	33 (17.7%)
RF missing (%)	12 (2.9%)	4 (2.1%)
Smoker (%)	99 (24.0%)	41 (21.9%)
Consumption of alcohol (%)	243 (59.0%)	108 (57.8%)
Mean BMI (SD)	25.0 (5.0)	24.7 (4.4)
Missing BMI (%)	29 (7.0%)	19 (10.2%)
Mean Rau score (SD)	NA	35.5 (35.4)
Missing Rau score (%)	NA	55 (29.4%)
Mean DAS28-esr score (SD)	4.0 (1.5)	4.1 (1.5)
Missing DAS28-esr score (%)	34 (8.3%)	22 (11.8%)
Mean infliximab dose per day (SD) (mg)	5.9 (7.7)	3.5 (3.5)
Missing infliximab dose per day (%)	107 (26.0%)	53 (28.3%)
csDMARD use (%)	343 (83.3%)	152 (81.3%)
Prednisone use (%)	229 (55.6%)	98 (52.4%)
Mean prednisone dose (SD) (mg)	5.7 (6.9)	NA
Other pain/anti-inflammatory drug use (%)	88 (21.4%)	26 (13.9%)
Postmenopausal (%)	113 (27.4%)	42 (22.5%)
Osteoarthritis (%)	45 (10.9%)	17 (9.1%)
Fibromyalgia (%)	28 (6.8%)	9 (4.8%)
Hypertension (%)	60 (14.6%)	18 (9.6%)
Diabetes (%)	14 (3.4%)	4 (2.1%)
Hyperlipidaemia (%)	17 (4.1%)	4 (2.1%)
Cardiac disorders (%)	28 (6.8%)	6 (3.2%)
Depression/anxiety (%)	47 (11.4%)	17 (9.1%)

BMI, body mass index; csDMARD, conventional synthetic disease modifying antirheumatic drug; DAS28-esr, disease activity measurement using 28 joints and erythrocyte sedimentation rate; HAQ, Health Assessment Questionnaire; NA, not available; p25, percentile 25; p75, percentile 75; RF, rheumatoid factor.

Table 2 depicts the results of the longitudinal analysis of the association between changes in BMI and changes in mean DAS28-esr (cohort 1), overall, and stratified by BMI categories. At cohort entry, 66 patients (16%) were obese, 114 (28%) overweight, 204 (50%) normal weight and 28 (7%) underweight. We did not observe significant changes of mean DAS28-esr given a change in BMI, neither overall nor in BMI categories. Yet, we observed a slight trend of decreasing BMI coefficients with decreasing BMI category. In underweight patients, an increase of 1 BMI unit resulted in a non-significant decrease of 0.14 in mean DAS28-esr (adjusted coefficient of BMI: −0.14, 95% CI −0.28 to 0.01), where the decrease diminished to −0.04 (95% CI −0.08 to 0.01) in normal weight patients, to −0.02 (95% CI −0.08 to 0.04) in overweight patients, and levelled out to 0.00 (95% CI −0.05 to 0.05) in obese patients. Furthermore, across all categories, crude and adjusted coefficients were highly similar to each other. Also adjusting for continuous prednisone dose instead of binary prednisone use had no impact on the effect estimate. Overall, all adjusted coefficients of BMI were 0.00 (−0.02 to 0.02). Furthermore, prednisone use had no effect modifying qualities (online supplemental material 6).

**Table 2:** Results from the longitudinal assessment of BMI and mean DAS28-esr changes (cohort 1) using crude and adjusted generalised estimation equations analyses overall and stratified by BMI category

	Overall n=412	Underweight n=28	Normal weight n=204	Overweight n=114	Obese n=66
Outcome: DAS28-esr	Coefficient of BMI (95% CI)	Coefficient of BMI (95% CI)	Coefficient of BMI (95% CI)	Coefficient of BMI (95% CI)	Coefficient of BMI (95% CI)
DAS28-esr	0.01 (−0.03 to 0.01)	0.12 (−0.26 to 0.03)	0.06 (−0.11 to −0.01)	0.04 (−0.10 to 0.03)	0.01 (−0.04 to 0.06)
DAS28-esr adjusted for time	0.00 (−0.02 to 0.02)	0.10 (−0.24 to 0.05)	0.04 (−0.08 to 0.01)	0.02 (−0.08 to 0.05)	0.00 (−0.05 to 0.05)
DAS28-esr adjusted for INF daily dose, time	0.00 (−0.02 to 0.02)	0.10 (−0.25 to 0.04)	0.04 (−0.08 to 0.01)	0.02 (−0.08 to 0.05)	0.00 (−0.05 to 0.05)
DAS28-esr adjusted for INF daily dose, age, sex, time*	0.00 (−0.02 to 0.02)	0.14 (−0.28 to 0.01)	0.03 (−0.08 to 0.01)	0.02 (−0.08 to 0.04)	0.00 (−0.05 to 0.05)
DAS28-esr adjusted for INF daily dose, age, sex, RF, smoking, menopause, osteoarthritis, fibromyalgia, hypertension, cardiac disorders, depression/anxiety, time	0.00 (−0.02 to 0.02)	0.14 (−0.28 to 0.01)	0.04 (−0.08 to 0.01)	0.03 (−0.09 to 0.04)	0.01 (−0.04 to 0.06)
DAS28-esr adjusted for INF daily dose, age, sex, RF, smoking, menopause, osteoarthritis, fibromyalgia, hypertension, cardiac disorders, depression/anxiety, and prednisone use, and other pain/anti-inflammatory drug use, time	0.00 (−0.02 to 0.02)	0.13 (−0.28 to 0.01)	0.04 (−0.08 to 0.01)	0.03 (−0.09 to 0.04)	0.01 (−0.04 to 0.06)
*Sensitivity analysis*: DAS28-esr adjusted for INF daily dose, age, sex, RF, smoking, menopause, osteoarthritis, fibromyalgia, hypertension, cardiac disorders, depression/anxiety, and prednisone dose, and other pain/anti-inflammatory drug use, time	0.00 (−0.02 to 0.01)	0.13 (−0.28 to 0.02)	0.04 (−0.08 to 0.01)	0.04 (−0.10 to 0.03)	0.01 (0.05 to 0.06)

*Main model.

BMI, body mass index; DAS28-esr, disease activity measurement using 28 joints and erythrocyte sedimentation rate; INF, infliximab; RF, rheumatoid factor.

Table 3 depicts the results of the longitudinal analysis of the association between changes in BMI and changes in mean Rau scores (cohort 2), overall, and stratified by BMI categories. At cohort entry, 28 patients (15%) were obese, 55 (29%) overweight, 94 (50%) normal weight and 10 (5%) underweight (the latter subgroup had too few patients for an analysis). We observed significant decreases in mean Rau scores given an increase of 1 BMI unit in the overall population in all models. The crude coefficient of BMI was −0.89 (95% CI −1.77 to −0.01). Adjustment for infliximab dose, age, sex and follow-up time emphasised the result, mean Rau scores would decrease by 1.05 given an increase of 1 BMI unit (coefficient of −1.05, 95% CI −1.92 to −0.19). Further adjusting did not change the coefficient more. Results among BMI categories were statistically non-significant given large CIs. In a post-hoc analysis without the underweight group, results were statistically non-significant.

**Table 3:** Results from the longitudinal assessment of BMI and mean Rau score changes (cohort 2) using crude and adjusted generalised estimation equations analyses overall and stratified by BMI category

	Overall* n=187	Overall except underweight* n=177	Normal weight n=94	Overweight n=55	Obese n=28
Outcome: Rau score	Coefficient of BMI (95% CI)	Coefficient of BMI (95% CI)	Coefficient of BMI (95% CI)	Coefficient of BMI (95% CI)	Coefficient of BMI (95% CI)
Rau score	0.89 (−1.77 to −0.01)	0.55 (−1.40 to 0.31)	0.38 (−1.98 to 1.22)	0.16 (−2.46 to 2.13)	0.44 (−2.15 to 1.28)
Rau score adjusted for time	0.91 (−1.79 to −0.03)	0.58 (−1.43 to 0.28)	0.38 (−2.01 to 1.25)	0.45 (−2.77 to 1.87)	0.14 (−1.92 to 1.65)
Rau score adjusted for DAS28-esr, time	0.91 (−1.79 to −0.03)	0.58 (−1.43 to 0.28)	0.38 (−2.00 to 1.24)	0.43 (−2.76 to 1.89)	0.15 (−1.92 to 1.63)
Rau score adjusted for INF daily dose, age, sex, time†	1.05 (−1.92 to −0.19)	0.78 (−1.62 to 0.07)	0.41 (−2.12 to 1.31)	0.59 (−2.94 to 1.76)	0.22 (−1.96 to 1.51)
Rau score adjusted for INF daily dose, age, sex, RF, smoking, menopause, osteoarthritis, fibromyalgia, hypertension, cardiac disorders, depression/anxiety, time	1.08 (−1.94 to −0.22)	0.79 (−1.64 to 0.05)	0.52 (−2.21 to 1.17)	0.41 (−2.77 to 1.94)	0.54 (−2.34 to 1.26)
Rau score adjusted for INF daily dose, age, sex, RF, smoking, menopause, osteoarthritis, fibromyalgia, hypertension, cardiac disorders, depression/anxiety, and prednisone use, and other pain/anti-inflammatory drug use, time	1.07 (−1.93 to −0.21)	0.78 (−163 to 0.06)	0.50 (−2.19 to 1.20)	0.34 (−2.68 to 2.00)	0.52 (−2.37 to 1.34)

*The underweight group with only 10 patients was too small for an assessment.

†Main model.

BMI, body mass index; DAS28-esr, disease activity measurement using 28 joints and erythrocyte sedimentation rate; INF, infliximab; RF, rheumatoid factor.

## Discussion

Our longitudinal investigation among RA patients in the SCQM until 2020 during their infliximab course of around 4.5 years yielded no association between changes in BMI and changes in mean DAS28-esr. We observed the most extreme result in underweight patients, where an increase of one BMI unit resulted in a non-significant decrease of 0.14 in mean DAS28-esr. Conversely, a unit increase in BMI was associated with a decrease of 1.05 in mean Rau scores. However, results were statistically non-significant across BMI categories, likely due to decreased sample size. For both cohorts, varying the adjusting variables did not change the results largely suggesting little influence of covariates on investigated associations.

Another recent study observed no difference in RA disease activity measure (Crohn’s disease activity index) over time in all BMI categories, as well as when comparing obese and non-obese patients.^20^ The study further reports worse outcomes for obese patients when assessing disease activity as a categorical variable though. While the evidence of obese patients having higher disease activity is frequent, its results originate mainly from cross-sectional evidence, and the use of categorical measures.^11^ Thus, it seems that the published evidence of patients with a higher BMI experiencing worse RA disease activity may be different from our assessment which assessed the influence of changes in BMI on changes in RA outcomes. The adipose tissue as driver of inflammation, in particular visceral fat emerged as the main driver in poor RA outcomes,^21^ but needs time to establish itself first. Thus, our study results may suggest that an increase in BMI may not be as bad as long-established obesity with regards to RA disease activity.

Weight cannot only increase, but also decrease. For example, a recent single blind trial reported a decrease from 5.2 to 4.2 in DAS28-esr among 40 RA patients who lost on average 9.5 kg during 12 weeks.^22^ Their finding suggests a short-term benefit from weight loss in patients with a high disease activity which is in contrast to our null result. Our descriptive trajectories in BMI and DAS28-esr suggest that there was sufficient variability in the variables to detect a potential association. Yet, it may be that our long-term observation with a mean follow-up of 4.5 years may have masked any potential short-term benefits or negative influences of a decrease or increase in BMI.

Our non-significant result of a slight decrease in mean DAS28-esr with increasing BMI in underweight patients adds to current literature suggesting a decreased antirheumatic treatment retention, and potentially poorer outcomes in underweight patients.^23 24^ While the low BMI may be intrinsic to other circumstances such as bad health and severe RA, our results may suggest that a slight increase in BMI may decrease RA disease activity. Although bigger studies with equally thorough methodology are needed to confirm this finding.

We observed an increase of one BMI unit is associated with a decrease of around one in mean Rau scores. This finding concurs with current literature suggesting less radiographic progressions in obese patients when compared with non-obese.^10^ Our results suggested a decrease in mean radiographic progression with increasing BMI for the entire population, not only obese patients whose results were non-significant (likely due to small sample size). Moreover, since the estimates of a linear GEE may be comparable with that of mixed effect models,^16^ we may want to consider what our results mean for an individual (instead of the population). At cohort entry on a mean Rau score of 35 (out of a maximum of 132), our population experienced on average a radiographic joint damage grade 2 (out of 5). Thus, we hypothesise a partially potentially reversible damage. Rau scores are mainly based on erosions and includes joint space narrowing as a concomitant feature.^14^ Rau and Herborn reported healing phenomena of erosions in RA,^25^ and were followed by more case reports.^26 27^ A recent study adds that a potential pathway could be through less osteitis among patients with high BMI.^28^ Thus, the interpretation of our findings in light of existing literature could be that, with increasing BMI, radiographic progression decreases (existing literature observed their binary correlation while we observed their moderate association).

Our study has several strengths, for example, the longitudinal assessment of continuous exposure and outcome variables able to account for fluctuations over time and which allowed assessing temporality and causality. Talking about evidence generation, we agree with Baker *et al* that ‘[a]n important limitation in studying obesity in this context is the difficulty differentiating mediators of the relationship between obesity and disease activity and response to therapy’.^20^ Thus, we tried our best to get beyond this limitation by drafting a DAG, imputing missing values accounting for repeated measures, and investigating both disease activity and radiographic erosions simultaneously, and by assessing the influence of confounders and/or mediators in various models. To fully control for confounders which may be mediators at the same time, g-estimation of structural nested models are needed, which, however, to our knowledge, do not accommodate continuous exposure or outcome measures.^29^ Yet, in our models, we did not observe a large influence of covariates which may imply little influence of a potentially misspecified model when adjusting for confounders and/or mediators. Inclusive assessment of all DMARDs was performed in a previous approach but model convergence was not achieved. It would have been interesting to derive the influence of the various agent and dosing. Hopefully, new models and increasing sample sizes may accommodate for such a study in the future. Thus, to form a study population using weight-dosed infliximab helped us to diminish the influence on various agents and dosing and likely yielded a high internal validity. Our homogeneous study population further had a rather long survival on infliximab with a mean of 4.5 years, including frequently measured BMI values because of its weighted dosing (≤10% of visits had missing BMI values). While limiting our study population to infliximab users may decrease generalisability of results, we observed that the characteristics of our population was comparable to those of all bDMARD initiators and other DMARD users in prior studies in SCQM.^30–32^ Thus, our results may be generalisable to all DMARD users in SCQM. The small sample size was a limitation in our study which may have led to many non-significant results. A further limitation was the low level of value completeness of just 20% in cohort 2, which may have hampered our results on the assessment of BMI and radiographic progression changes. Yet a powerful multiple imputation process was deployed running 80 iterations which took into account repeated measures of individual patients, and given the inclusion criteria, individuals were only missing a few measurements of exposure or outcome, never all of them.

## Conclusions

Our longitudinal investigation in the SCQM until 2020 suggests that BMI changes not necessarily lead to changes in mean DAS28-esr. Yet a non-significant decrease in mean DAS28-esr of 0.14 with every unit increase in BMI in underweight patients may yield insights into this difficult RA phenotype. Furthermore, a decrease of mean Rau scores of 1.05 with every unit increase in BMI in the overall population may suggest a protective effect not limited to the obese population. Yet, the small sample size warrants further investigation in bigger cohorts to corroborate our findings.

## Data Availability

Data are available upon reasonable request. The data analysed in this study is available from the corresponding author upon reasonable request and after having received approval from the license holder (SCQM).
