# Supplementary material for: Longitudinal associations between body mass index and changes in disease activity and radiographic progression in rheumatoid arthritis patients treated with infliximab
Source: RMD Open. 2023 Oct 6;9(4):e003396. doi: 10.1136/rmdopen-2023-003396 (PMC10565266; doi:10.1136/rmdopen-2023-003396)
Supplement: Supplementary data [file rmdopen-2023-003396supp001.pdf]

Supplementary Material 1. Additional information on data standardization and harmonization

- The SCQM database is populated via standardized online case report forms (CRFs) filled out by rheumatologists or health care professionals (HCP) trained for the purpose by the SCQM, where a series of conditions are implemented to ensure data plausibility and consistency (e.g. for dates and lab values). The SCQM online database has an audit trail and a database user manual is made available to all users.
- Remote Monitoring: semi-automated checks are carried out (at least) on a quarterly basis and in the event of other data inconsistencies, a dedicated data manager from the SCQM gets in touch with the treating rheumatologist/HCP to clarify and correct the data. The SCQM data managers also work with queries sent to rheumatologists and regularly follow up on them. Via the mySCQM patient app, patients are also able to view the current status of their medication (as recorded in the SCQM database) and provide feedback on inconsistent data to the data manager who then acts upon this information.
- Central Data Monitoring: the SCQM gets in touch with institutions with rheumatologists and HCPs contributing data to the SQM database twice per year to make sure everything is clear, that everyone is up to date and to provide trainings as required.

Supplementary Material 2. Variable missingness

Variable missingness was assessed separately for each cohort and the multiple imputation procedure was carried out for each cohort separately.

Since we had longitudinal data with repeated observations per patient we used BLIMP software 2.2 which allows for multi-level imputation. We carried out 80 imputations in all cohorts because datasets were small. A potential scale reduction (PSR) factor of <1.05 suggests convergence. In order to achieve convergence of the model, we had to simplify the model by deleting categorical variables with high missingness (e.g. alcohol consumption).

Supplementary Table 1. Variable missingness per cohort and potential scale reduction in the multiple imputation

	**Cohort 1**	**Cohort 2**
**Complete information**	55.8%	18.2%
**Missingness of DAS28-esr**	8.3%	11.8%
**Missingness of Rau scores**	NA	29.4%
**Missingness of dose**	26.0%	28.3%
**Missingness of RF**	2.9%	2.1%
**Missingness of BMI**	7.0%	10.2%
**PSR**	<1.01	<1.02

BMI: body mass index, DAS28-esr: disease activity measurement using 28 joints and erythrocyte sedimentation rate, NA: not available, PSR: potential scale reduction, RF: rheumatoid factor

Supplementary Material 3. Covariates assessed at cohort entry and during longitudinal assessment including variable type, lookback window, and time-varying status

Supplementary Table 2. Covariates assessed at cohort entry and during longitudinal assessment including variable type, lookback window, and time-varying status

Variables	Type	Lookback window	Time-varying	Covariate included in the final model
Follow-up time	continuous	none	yes	yes
Age	continuous	ever before	no	yes
Sex	Binary	ever before	no	yes
Smoking*	Binary	ever before	yes	yes
Alcohol consumption*	Binary	ever before	yes	no
DAS28-esr	continuous	at cohort entry	yes	cohort 2 yes
Rheumatoid factor	Binary	ever before	no	yes
Infliximab dose per day	continuous	at cohort entry	yes	yes
Conventional synthetic DMARDs	Binary	at cohort entry	yes	no
Prednisone use	Binary	at cohort entry	yes	yes
Other pain/anti-inflammatory medication use	Binary	at cohort entry	yes	yes
Postmenopausal state ͳ	Binary	ever before	yes	yes
Osteoarthritis or arthroplasty ͳ	Binary	ever before	yes	yes
Fibromyalgia ͳ	Binary	ever before	yes	yes
Hypertension (diagnosis or treatment) ͳ	Binary	ever before	yes	yes
Diabetes (diagnosis or treatment) ͳ	Binary	ever before	yes	no
Hyperlipidemia (diagnosis or treatment) ͳ	Binary	ever before	yes	no
Cardiac disorders (diagnosis or treatment) ͳ	Binary	ever before	yes	yes
Depression / Anxiety ͳ	Binary	ever before	yes	yes

DAS28-esr: rheumatoid arthritis disease activity measurement including 28 joints and erythrocyte sedimentation rate; DMARD: disease-modifying antirheumatic drug
* values were carried forward if there was no value in the subsequent visits
ͳ if there was ever a positive entry, this entry was carried forward because considered chronic

Supplementary Material 4: Study composition

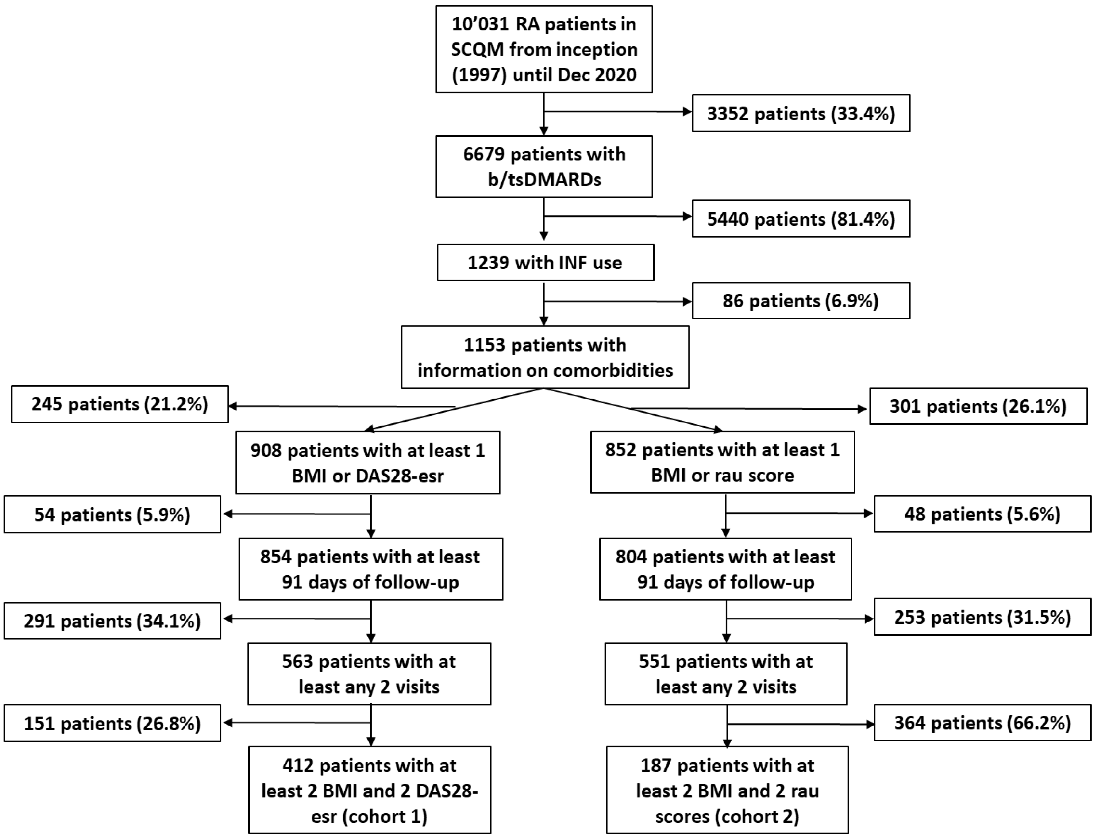

Supplementary Figure 1. Study composition of cohort 1 and cohort 2
BMI: body mass index; b/tsDMARDs: biologic or targeted synthetic disease modifying anti rheumatic drugs; DAS28-esr: rheumatoid arthritis disease activity measurement using 28 joints and erythrocyte sedimentation rate; INF: infliximab; RA: rheumatoid arthritis; SCQM: Swiss Clinical Quality Management of Rheumatic Diseases registry

Supplementary Material 5: BMI, DAS28-esr and Rau score trajectories over the follow-up time

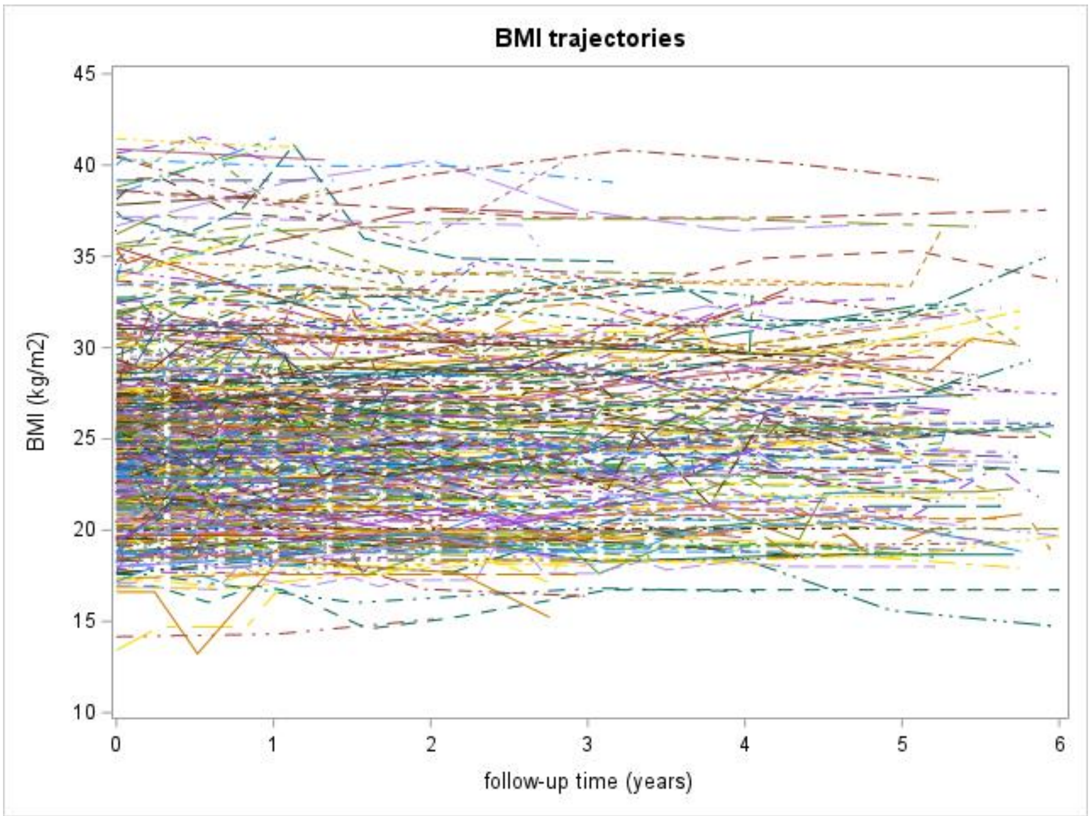

Supplementary Figure 2. BMI trajectories of cohort 1, multiple imputation run 1
The individual lines show BMI values of individual patients over their follow-up. The plot’s follow-up time was trimmed at 6 years for better visibility.
BMI: body mass index;

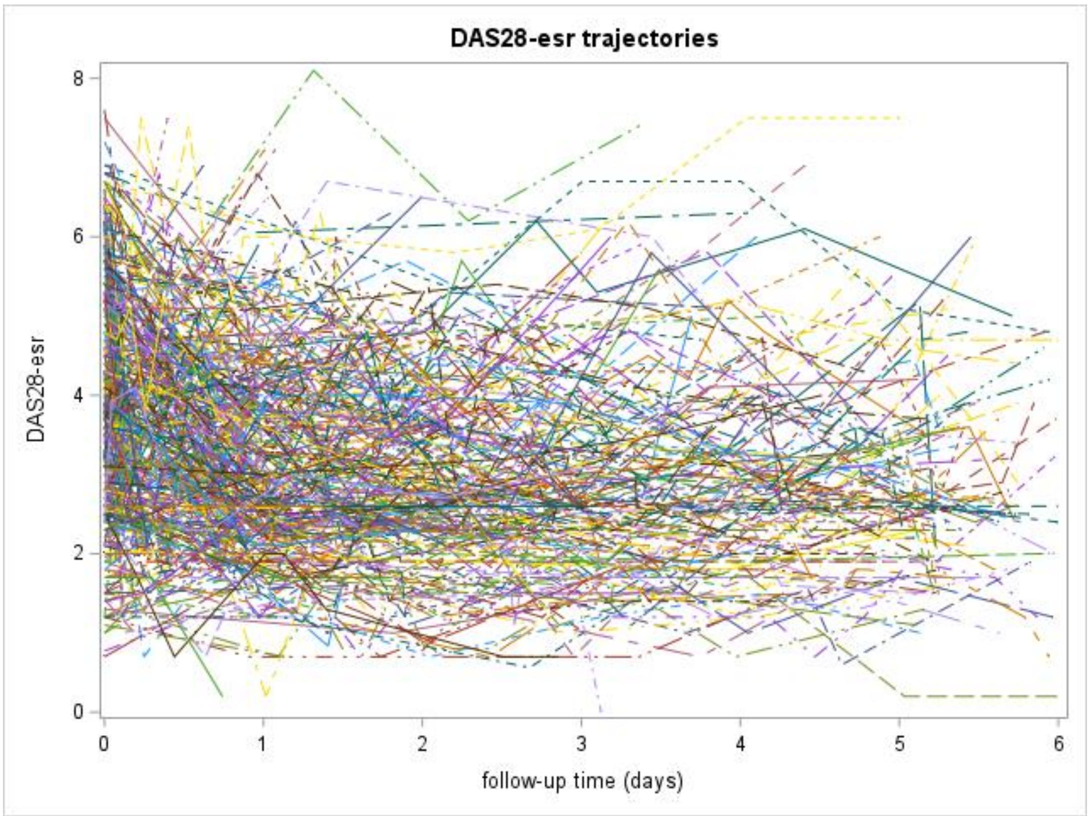

Supplementary Figure 3. DAS28-esr score trajectories of cohort 1, multiple imputation run 1
The individual lines show values of DAS8-esr of individual patients over their follow-up. The plot's follow-up time was trimmed at 6 years for better visibility.
DAS28-esr: rheumatoid arthritis disease activity measurement using 28 joints and erythrocyte sedimentation rate;

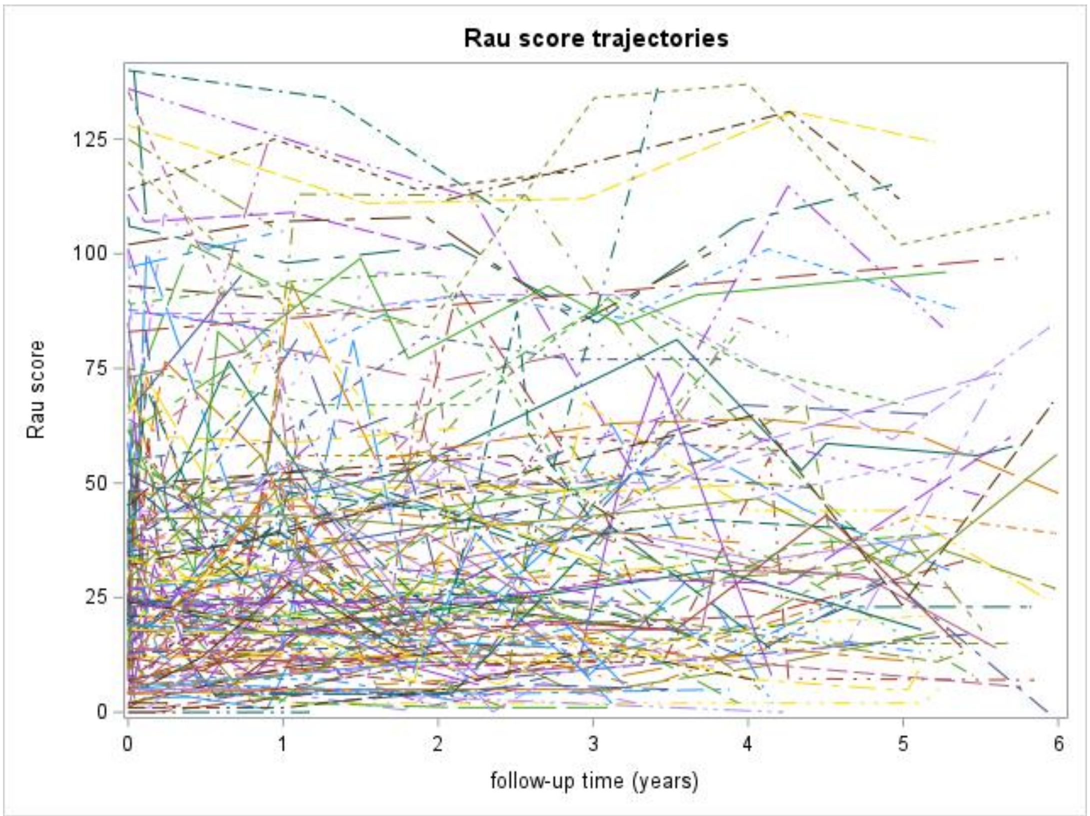

Supplementary Figure 4. Rau score trajectories of cohort 1, multiple imputation run 1
The individual lines show Rau scores of individual patients over their follow-up. The plot's follow-up time was trimmed at 6 years for better visibility.

Supplementary Material 6. Results from the sensitivity analysis in which prednisone use was tested for effect modifier qualities in the assessment of BMI and mean DAS28-esr changes (cohort 1)

Supplementary Table 3. Results from the longitudinal assessment of BMI and mean DAS28-esr changes (cohort 1) using crude and adjusted GEE analyses stratified by prednisone use at index date

	**Prednisone at index date n = 229**	**No prednisone at index date n = 183**
**Outcome: DAS28-esr**	Coefficient of BMI (95% CI)	Coefficient of BMI (95% CI)
DAS28-esr	0.00 (-0.03 – 0.02)	-0.01 (-0.04 – 0.02)
DAS28-esr adjusted for time	0.00 (-0.02 – 0.03)	0.00 (-0.03 – 0.02)
DAS28-esr adjusted for INX daily dose, time	0.00 (-0.02 – 0.03)	0.01 (-0.03 – 0.02)
**DAS28-esr adjusted for INX daily dose, age, sex, time***	**0.00 (-0.02 – 0.03)**	**0.01 (-0.03 – 0.02)**
DAS28-esr adjusted for INX daily dose, age, sex, RF, smoking, menopause, osteoarthritis, fibromyalgia, hypertension, cardiac disorders, depression/anxiety, time	0.00 (-0.02 – 0.03)	0.01 (-0.03 – 0.02)
DAS28-esr adjusted for INX daily dose, age, sex, RF, smoking, menopause, osteoarthritis, fibromyalgia, hypertension, cardiac disorders, depression/anxiety, and other pain/anti-inflammatory drug use, time	0.00 (-0.02 – 0.02)	0.01 (-0.03 – 0.02)

BMI: body mass index, DAS28-esr: disease activity measurement using 28 joints and erythrocyte sedimentation rate, INX: infliximab, RF: rheumatoid factor
*main model
